# A Low Cost Aqueous Zn–S Battery Realizing Ultrahigh Energy Density

**DOI:** 10.1002/advs.202000761

**Published:** 2020-10-20

**Authors:** Wei Li, Kangli Wang, Kai Jiang

**Affiliations:** ^1^ State Key Laboratory of Advanced Electromagnetic Engineering and Technology School of Electrical and Electronic Engineering Huazhong University of Science and Technology Wuhan 430074 P. R. China

**Keywords:** aqueous Zn–S batteries, conversion reaction, high capacity, high energy density, sulfur cathodes

## Abstract

Rechargeable aqueous zinc ion batteries are enabled by the (de)intercalation chemistry, but bottlenecked by the limited energy density due to the low capacity of cathodes. In this work, carbon nanotubes supported 50 wt% sulfur (denoted as S@CNTs‐50), as a conversional cathode, is employed and a high energy density aqueous zinc–sulfur (Zn–S) battery is constructed . In the electrolyte of 1 m Zn(CH_3_COO)_2_ (pH = 6.5) with 0.05 wt% I_2_ additive where I_2_ can serve as medium of Zn^2+^ ions to reduce the voltage hysteresis of S@CNTs‐50 and stabilize Zn stripping/plating, S@CNTs‐50 delivers a high capacity of 1105 mAh g^−1^ with a flat discharge voltage of 0.5 V, realizing an energy density of 502 Wh kg^−1^ based on sulfur, which is one of the highest values reported in aqueous Zn‐based batteries that use mild electrolyte. Moreover, the chemical materials cost of this aqueous Zn–S battery can be lowered to be $45 kWh^−1^ due to the cheap raw materials, reaching to the level of pumped energy storage. Ex situ X‐ray diffraction, Raman spectra, X‐ray photoelectron spectrum, and transmission electron microscopy measurements reveal that sulfur cathode undergoes a conversion reaction between S and ZnS.

## Graphical abstract

An aqueous zinc–sulfur (Zn–S) battery based on carbon nanotubes supported sulfur (S@CNTs‐50) cathode is developed. Due to the optimized electrolyte, additive, and sulfur content, S@CNTs‐50 realizes high energy density over 500 Wh kg^−1^ through a conversion reaction from S to ZnS, exceeding most of reported Zn‐based aqueous batteries.

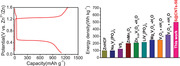

Rechargeable batteries using green, nonflammable, and nontoxic electrolyte are highly demanded with increasing concerns about the safety issues.^[^ ^1^, ^2^, ^3^, ^4^, ^5^, ^6^ ^]^ Although aqueous batteries are well satisfying the above criteria, the energy density obtained at present is far below that of nonaqueous systems, such as Li‐ion and Li–S batteries.^[^ ^7^, ^8^, ^9^ ^]^ Due to the limitation of electrochemical window, the cell voltages of aqueous batteries are not easy to reach the level of Li‐ion batteries. Therefore, exploring electrodes with high capacity is critical to achieve high energy density. In this regard, Zn metal anode holds great promise because Zn possesses the merits of high theoretical capacity (810 mAh g^−1^; 5855 mAh cm^−3^), low cost (2 US$ kg^−1^), proper potential (−0.76 V vs standard hydrogen electrode), and high conductivity. The early alkaline Zn‐based batteries, such as Zn–MnOOH and Zn–NiOOH, displayed high voltage and high energy density but suffered from poor cyclability due to the severe corrosion, Zn dendrites, water decomposition, and active material dissolution.

Recently, the state‐of‐art rechargeable aqueous Zn‐based batteries are enabled by the (de)intercalation chemistry with mild electrolyte, rendering the rapid development of aqueous Zn‐ion batteries.^[^ ^10^, ^11^, ^12^, ^13^, ^14^ ^]^ Intercalated cathodes, such as MnO_2_,^[^ ^15^, ^16^, ^17^, ^18^, ^19^, ^20^, ^21^, ^22^, ^23^ ^]^ phosphate,^[^ ^24^, ^25^, ^26^ ^]^ vanadates,^[^ ^27^, ^28^, ^29^, ^30^, ^31^, ^32^, ^33^, ^34^, ^35^, ^36^, ^37^, ^38^, ^39^, ^40^ ^]^ Prussian blue,^[^ ^41^, ^42^ ^]^ and polymers,^[^ ^43^, ^44^, ^45^ ^]^ show good cycling stability but a relatively low energy densities in the range from 50 to 300 Wh kg^−1^ (for cathode). In the pursuit of higher energy density, cathodes with conversion mechanism are very desirable because they are expected to have higher capacity over 500 mAh g^−1^.^[^ ^46^, ^47^, ^48^, ^49^, ^50^ ^]^ As a typical conversion mechanism and low cost raw material, sulfur (0.25 US$ kg^−1^) holds high theoretical capacity up to 1675 mAh g^−1^, which will be very attractive to achieve a high energy density over 300 Wh kg^−1^ if it can be used as a cathode for aqueous Zn‐based battery.

Herein, we design a rechargeable and low cost aqueous Zn–S battery using carbon nanotubes supported 50 wt% sulfur (S@CNTs‐50) as cathode. It is found that the pH values and additive of electrolytes significantly affect the reversible capacity and voltage hysteresis of S@CNTs‐50. When 1 m Zn(CH_3_COO)_2_ (pH = 6.5) with 0.05 wt% I_2_ additives is used as electrolyte, where I_2_ acts as Zn^2+^ ion carrier to improve kinetics, S@CNTs‐50 realizes a high capacity of 1105 mAh g^−1^ with a small overpotential, achieving a high energy density of 502 Wh kg^−1^ (based on sulfur). Besides, owing to the low cost of raw materials, the chemical materials cost of this aqueous Zn–S battery can be lowered to $45 kWh^−1^, reaching to the level of pumped energy storage. Furthermore, the conversion mechanism between S and ZnS has been revealed by X‐ray diffraction (XRD), Raman, X‐ray photoelectron spectrum (XPS), and transmission electron microscopy (TEM) tests.

The preparation of carbon nanotubes supported sulfur (S@CNTs) composites was through a conventional molten infiltration method at 160 °C for 16 h. Since the pore volume of CNTs is 0.572 cm^3^ g^−1^, if all pores are filled by sulfur, the sulfur loading in S@CNTs can be calculated to 54 wt%. Thus, we prepared S@CNTs‐50 with sulfur loading of 50 wt%, as confirmed by thermogravimetry (TG) analysis (**Figure** **1**a). For comparison, we also synthesize three groups of composites with sulfur content of 30, 40, and 60 wt%, respectively (denoted as S@CNTs‐30, S@CNTs‐40, and S@CNTs‐60, respectively). After sulfur melt infiltration into CNTs, the intensity of sulfur diffraction peaks in all composites increases with the increase of sulfur content from 30 to 60 wt% (Figure 1b). Meanwhile, the N_2_ adsorption and desorption isotherm also discloses a great decrease of the Brunnauer–Emmett–Teller (BET) specific surface area from 364 to 70.8 m^2^ g^−1^ in S@CNTs‐50 (Figure 1c). The TEM image reveals that the CNTs show a smooth surface with a diameter of about 20 nm (Figure 1d). In contrast, sporadic particles in the inner pore of CNTs are observed in S@CNTs‐50, which is probably due to the penetration of sulfur (Figure 1e,f; and Figure S1, Supporting Information). The TEM image (Figure 1g) and mapping (Figure 1h,i) show that sulfur is generally homogeneous distributed over CNTs with a slight of sulfur particles anchored on the surface of CNTs.

**Figure 1:**
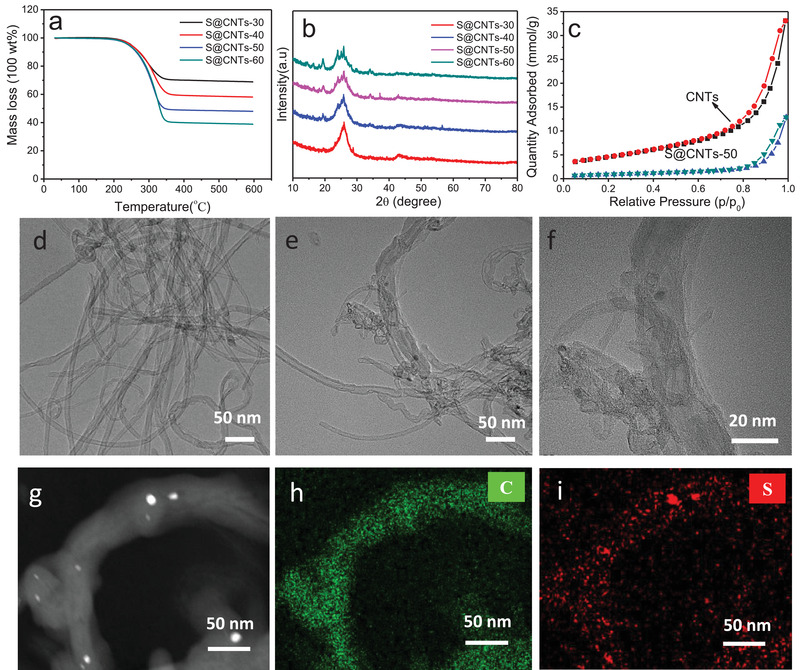
a) TG curves and b) XRD patterns of S@CNTs composites. c) N_2_ adsorption and desorption isothermal of S@CNTs‐50 and CNTs. TEM images of d) CNTs and e,f) S@CNTs‐50. g) HAADF TEM image and TEM mapping of h) C and i) S of S@CNTs‐50.

In order to examine the influence of electrolytes especially pH values on the electrochemical behavior of S@CNTs‐50 cathode, three easily accessible electrolytes are examined. In 1 m Zn(CF_3_SO_3_)_2_ (pH = 4.4), S@CNTs‐50 shows a discharge capacity of 412 mAh g^−1^ (on the basis of sulfur) and a charge capacity of 260 mAh g^−1^ in the first cycle at a current density of 100 mA g^−1^ (**Figure** **2**a), corresponding to an initial coulombic efficiency (CE) of 63%. In the second cycle, it only delivers a reversible capacity of 230 mAh g^−1^, indicating a low utilization of sulfur in S@CNTs‐50. Besides, a large voltage hysteresis of 1.1 V suggests the sluggish kinetics reaction of S@CNTs‐50 in Zn(CF_3_SO_3_)_2_ electrolyte. In 1 m ZnSO_4_ (pH = 5.2), S@CNTs‐50 releases a high initial discharge capacity of 995 mAh g^−1^ but a very low charge capacity of 254 mAh g^−1^ (Figure 2b), causing a low initial CE of 26%. Compared with 1 m Zn(CF_3_SO_3_)_2_, the voltage hysteresis of S@CNTs‐50 slightly decreases to 0.95 V in 1 m ZnSO_4_. In the case of 1 m Zn(CH_3_COO)_2_ (pH = 6.5), the initial reversible capacity and CE increase to 685 mAh g^−1^ and 98% (Figure 2c), respectively. However, the delivered capacity is far lower than the theoretical capacity of sulfur (1675 mAh g^−1^). Besides, the voltage hysteresis is as large as 0.9 V, leading to low energy efficiency, which is far from satisfactory.

**Figure 2:**
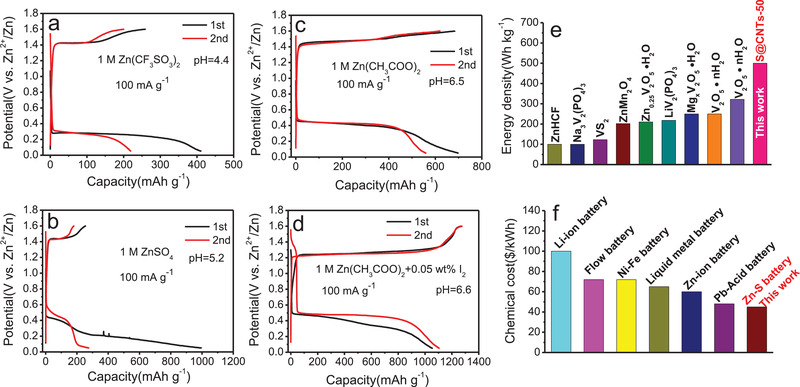
Charge and discharge curves of S@CNTs‐50 cathode in a) 1 m Zn(CF_3_SO_3_)_2_, b) 1 m ZnSO_4_, c) 1 m Zn(CH_3_COO)_2_, and d) 1 m Zn(CH_3_COO)_2_ with 0.05 wt% I_2_ as additive. e) Comparison of energy density of S@CNTs‐50 with reported cathode. f) The chemical materials cost of this aqueous Zn–S battery with other batteries.

In order to make full use of sulfur and reduce voltage hysteresis, the electrochemical behavior of S@CNTs‐50 in the electrolyte of 1 m Zn(CH_3_COO)_2_ with 0.05 wt% I_2_ as additive was also investigated. When the additive of 0.05 wt% I_2_ is added into 1 m Zn(CH_3_COO)_2,_ the color of electrolyte becomes yellow (Figure S2, Supporting Information). Surprisingly, S@CNTs‐50 shows a much higher reversible capacity of 1105 mAh g^−1^ with a flat discharge plateau of 0.5 V at 100 mA g^−1^ (Figure 2d). Given the capacity contribution from CNTs (Figure S3, Supporting Information), the capacity of sulfur can reach 1005 mAh g^−1^. Accordingly, S@CNTs‐50 cathode can realize a maximum energy density of 502 Wh kg^−1^ (based on the mass of sulfur). To our best knowledge, this is one of the highest values among recently reported aqueous Zn‐based batteries (Figure 2e). Furthermore, the voltage hysteresis of S@CNTs‐50 can be lowered to 0.72 V, demonstrating an improved kinetics in the uptake and removal of Zn^2+^ ions with the electrolyte additive of I_2_. Such case can be ascribed to the reason that I_2_ can be served as medium of Zn^2+^ ions and reduce the reaction barrier,^[^ ^51^, ^52^, ^53^ ^]^ which can be also supported by the small discharge/charge plateaus at 1.3 V corresponding to the redox reaction of I_2._ Besides, in other two electrolytes, I_2_ additive can also reduce the voltage gap (Figure S4, Supporting Information). When the content of I_2_ additive is increased to 0.1 wt% (Figure S5, Supporting Information), the discharge capacity can be increased to 1302 mAh g^−1^ due to the capacity contribution from I_2_, but the discharge plateau and overpotential show no obvious change, which implies that a higher content additive over 0.1 wt% exert little effect on reducing the overpotential of S@CNTs‐50. The single long charge and discharge plateau of this aqueous Zn–S battery, similar to the case of Al–S battery, suggests a solid‐state conversion reaction of S to ZnS.^[^ ^54^, ^55^ ^]^ The strikingly different behavior of S@CNTs‐50 in these electrolytes indicates that the neutral electrolytes are more favorable for S@CNTs‐50 to achieve high capacity and low voltage hysteresis, which is probably due to the improved stability of sulfides in neutral electrolytes because sulfides easily suffer disproportionated reactions in acid solution.^[^ ^56^ ^]^

Besides lowering the voltage hysteresis, the additive of 0.05 wt% I_2_ in 1 m Zn(CH_3_COO)_2_ can also stabilize Zn stripping and plating. As shown in Figure S6 (Supporting Information), the Zn symmetrical cells using 1 m Zn(CH_3_COO)_2_ with 0.05 wt% I_2_ additive show less fluctuation and smaller overpotential in galvanostatic curves than that of 1 m Zn(CH_3_COO)_2_. Moreover, due to the low cost of raw material, the cost of this aqueous Zn–S battery can be very low. Figure 2f compares the chemical materials cost of this aqueous Zn–S battery with reported other systems. The materials cost can be reduced to $45 kWh^−1^ (Table S1, Supporting Information), which is one of the lowest among the reported battery systems.^[^ ^57^ ^]^ Besides, the material cost of this aqueous Zn–S battery can be further reduced if other cheaper conducting carbon can be used. The high energy density and low cost of the aqueous Zn–S battery endow it as a cheap, green, safe, and high performance energy storage technology.

The influence of sulfur content on the electrochemical behavior of composites was investigated. As shown in Figure S7 (Supporting Information), the discharge capacity (on the basis of sulfur) decreases with the increase of sulfur content from 30 to 60 wt%. S@CNTs‐30 delivers the highest capacity of 1088 mAh g^−1^ and the smallest overpotential of 0.72 V at 200 mA g^−1^, but it suffers severe side reactions at 1.4 V in the charge process. When the sulfur content increases to 40 and 50 wt%, the capacities are 1013 and 991 mAh g^−1^ for S@CNTs‐40 and S@CNTs‐50, respectively, while the overpotentials for both electrodes are almost the same (0.8 V). If calculated based on the mass of composites (sulfur and CNTs), the capacity for S@CNTs‐50 (496 mAh g^−1^) is higher than that of S@CNTs‐40 (405 mAh g^−1^). A further increase of sulfur content to 60 wt% incurs a lower capacity of 659 mAh g^−1^ (395 mAh g^−1^ for composite) and a higher overpotential of 0.82 V. Therefore, taking into the comprehensive consideration, we select S@CNTs‐50 sample, which features a relatively high capacity and low overpotential.

The rate capacities and cycling performance of S@CNTs‐50 in 1 m Zn(CH_3_COO)_2_ with I_2_ as additives were further tested. S@CNTs‐50 can give a capacity of 1046 mAh g^−1^ at a current density of 400 mA g^−1^ (**Figure** **3**a). At elevated current densities of 1000, 2000, and 4000 mA g^−1^, capacities of 680, 550, and 407 mAh g^−1^ can be kept. Besides, the discharge plateau decreases from 0.5 to 0.2 V when the current density increases from 400 to 4000 mA g^−1^ (Figure 3b), indicating that the kinetics of reaction is sluggish. To improve the kinetics, the behavior of S@CNTs‐50 was evaluated at a higher temperature. Figure 3c compares the charge and discharge curves of S@CNTs‐50 cathode at 25 and 55 °C at a current density of 1000 mA g^−1^. The S@CNTs‐50 can give a capacity of 762 mAh g^−1^ at 25 °C with a voltage hysteresis of 0.96 V. In contrast, at 55 °C, S@CNTs‐50 exhibits a higher capacity of 1335 mAh g^−1^ with a smaller voltage hysteresis of 0.8 V, showing a great improvement in reversible capacity and kinetics at a high temperature. More importantly, using high performance catalyst is also favorable to improve electrochemical reaction. When 5 wt% Pt catalyst is added into S@CNTs‐50 cathode, it achieves a high capacity of 1193 mAh g^−1^ with a small voltage hysteresis of 0.6 V at 100 mA g^−1^ at 25 °C with high mass loading of 3.04 mg cm^−2^, reaching a high energy density of 634 Wh kg^−1^ based on sulfur (Figure S8, Supporting Information).

**Figure 3:**
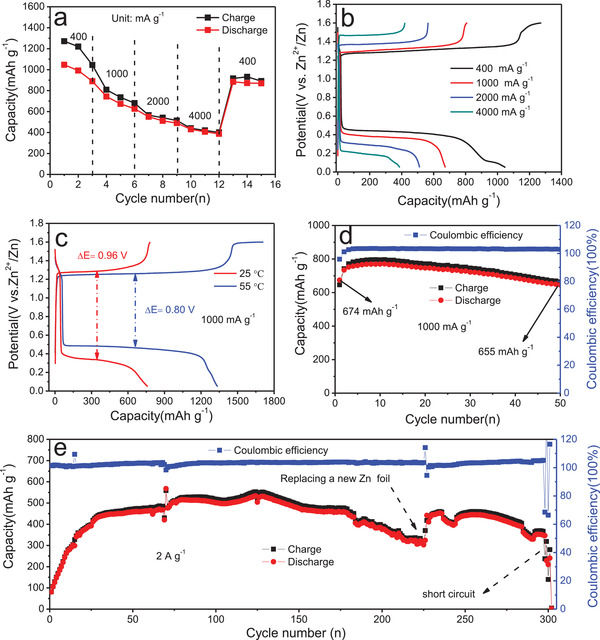
a) Rate capacities and b) charge and discharge profiles of S@CNTs‐50 at various current densities, c) Charge and discharge curves of S@CNTs‐50 at 25 and 55 °C at 1000 mA g^−1^, d) Cycling performance of S@CNTs‐50 cathode at a current density of 1000 mA g^−1^, e) long‐term cycling performance of S@CNTs‐50 cathode at 2000 mA g^−1^.

Figure 3d,e shows the cycling performance of S@CNTs‐50. An initial discharge capacity of 674 mAh g^−1^ is delivered at a current density of 1 A g^−1^, and slightly increases to 771 mAh g^−1^ after 11 cycles due to the activation process. After 50 cycles, the reversible capacity is kept at 655 mAh g^−1^, corresponding to capacity retention of 85%. At a high current density of 2 A g^−1^, the capacity of S@CNTs‐50 rapidly increases from 81 mAh g^−1^ in the first cycle to 435 mAh g^−1^ in the 30th cycle, and keeps steady at 458 mAh g^−1^ in the 177th, and decreases to 302 mAh g^−1^ after 225 cycles. When a fresh Zn anode is changed, the capacity of S@CNTs‐50 recovers to 450 mAh g^−1^, but it drops to zero due to the dendrite induced short circuit after 303 cycles. These results indicate that the capacity fading of S@CNTs‐50 is not only related with the dissolution of resultant because no obvious morphological and structural change is observed from the cycled S@CNTs‐50 electrode (Figure S9, Supporting Information),^[^ ^47^ ^]^ but also with the decomposition of electrolyte (Figure S10, Supporting Information), Zn dendrite and depletion (Figure S11, Supporting Information), which causes water loss, O_2_ generation and reduces the cycling efficiency. In fact, the Zn dendrite is fatal for the cycling of Zn–S battery. In our test process, some coin cells can only sustain less than 30 cycles due to the dendrite induced short circuit (Figure S12, Supporting Information). Compared with other reported cathodes for aqueous Zn‐based batteries, the cycling is not as attractive as them, but the energy density of S@CNTs‐50 is much higher (Table S2, Supporting Information).The influence of mass loading of sulfur on the discharge capacity of S@CNTs‐50 was also investigated. At a low loading of 0.7 mg cm^−2^, a capacity of 960 mAh g^−1^ can be released at a current density of 1 A g^−1^ (Figure S13, Supporting Information). When the mass loading is further increased to 2.5 mg cm^−2^, the S@CNTs‐50 cathode can still achieve a high capacity of 680 mAh g^−1^ and the discharge plateau almost keep unchanged, which is critical to develop high mass loading cathode for practical application. In addition, a pouch packing battery with four stacks was assembled to test its feasibility (Figure S14a–c, Supporting Information). The packing battery shows a discharge capacity of 0.33 Ah and an average discharge plateau of 0.4 V (Figure S14d, Supporting Information), corresponding to an energy density of 68 Wh kg^−1^ (based on the total mass of sulfur and CNTs in S@CNTs‐50). Considering the abundant, nontoxic and cheap raw materials (S, CNTs, and Zn(CH_3_COO)_2_), as well as its potential to raise the discharge voltage, the energy density of this system can be expected a much higher value.

Ex situ XRD, Raman, TEM, and XPS measurements were performed to illuminate the reaction mechanism. When S@CNTs‐50 is discharged to 0.4 V, indiscernible change can be observed from the XRD pattern compared with pristine S@CNTs‐50 (**Figure** **4**a). However, when S@CNTs‐50 is further discharged to 0.3 V, the peaks of S_8_ decrease and weaken, and three additional broad peaks at diffraction angle of 28°, 48°, and 56° appear, which matches well with the characterized peaks of ZnS (JCPDS No.05‐0566). At the fully discharged 0.05 V, the ZnS signal remains unchanged and the signal of S_8_ peaks almost vanishes. The discharge process demonstrates that S undergoes conversion reaction from S to ZnS (Equation (1)). In the charge process, both signals of S and ZnS can be observed at the 1.2 V (Figure S15a, Supporting Information), and the signals of ZnS weaken at 1.25 V (Figure S15b, Supporting Information). At the fully charged 1.6 V, strong S_8_ peaks reappear with the disappearance of ZnS, indicating the reversible conversion from ZnS to S. Such conversion reactions are also supported by the evolution of Raman spectra (Figure 4b). Obvious signals of ZnS at 182, 316, 371, and 423 cm^−1^ appear at the fully discharged state along with the strength decrease of S at 218 and 475 cm^−1^, indicating the conversion of S to ZnS. When the electrode is charged to 1.6 V, the peaks of ZnS almost disappear along with the reappearance of S peak at 218 cm^−1^, indicating the conversion of ZnS to S, which are consistent with the XRD results in Figure 4a.

**Figure 4:**
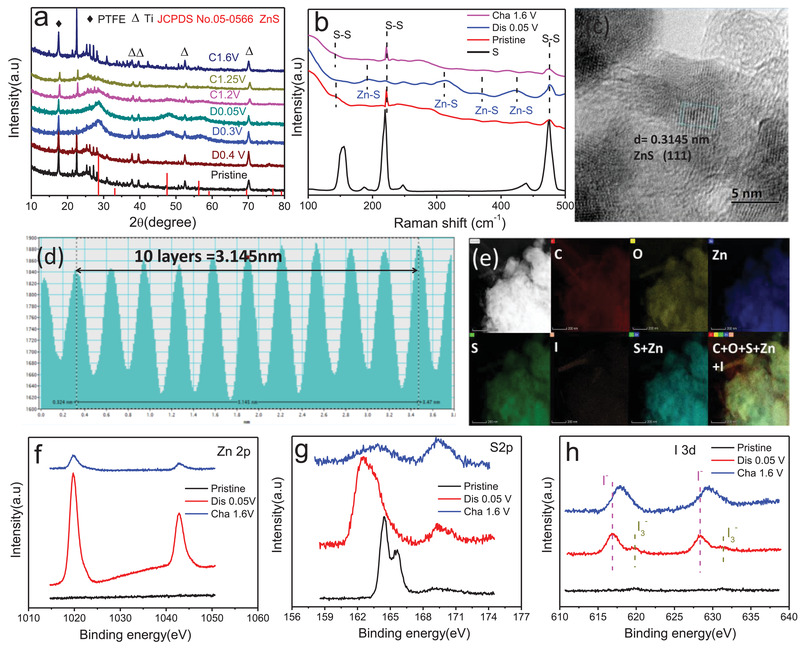
a) XRD patterns and b) Raman spectra of S@CNTs‐50 at different states. c,d) TEM images and e) TEM mapping of S@CNTs‐50 at fully discharged state. XPS spectra of f) Zn2p, g) S2p, and h) I3d at the pristine, fully discharged and charged state.

Further evidence can be also obtained from the TEM image and elemental mapping of the fully discharged S@CNTs‐50 electrode. The active materials show obvious volume expansion (from S to ZnS) and aggregation (Figure S16, Supporting Information) compared with the pristine S@CNTs‐50. The interlayer spacing of discharged product is measured to be 0.314 nm (Figure 4c,d), matching well with the (111) lattice plane of ZnS (JCPDS No.05‐0566), confirming that the discharge product of S@CNTs‐50 is ZnS. Moreover, Zn and S elemental mapping can be clearly observed from the TEM mapping (Figure 4e) except for C, O and I, and Zn mapping coincides well with that of S, which also demonstrates the formation of ZnS.

XPS was further conducted to check the surface change of electrode before and after cycling. Two strong Zn peaks at 1020 and 1043 eV appear when S@CNTs‐50 is fully discharged to 0.05 V and they drastically weakens when S@CNTs‐50 is fully charged to 1.6 V (Figure 4f), indicating the electrochemical insertion and extraction of Zn. Meanwhile, obvious S^2−^ signal at 160 eV is detected at the fully discharged 0.05 V (Figure 4g). When S@CNTs‐50 is charged to 1.6 V, the signal intensity of S is much weak, while the SO_4_^2−^ becomes strong, which suggests that part of surface S can be further oxidized into SO_4_^2−^. This result is also consistent with the significant intensity increase of O signal in S@CNTs‐50 at fully charged state (Figure S17, Supporting Information). Such case also reveals the possible capacity degradation mechanism of S@CNTs‐50 because SO_4_^2−^ can be hardly utilized again once it forms, reducing the electrode reversibility. Meanwhile, the SO_4_^2−^ can be further dissolves in aqueous electrolyte and causes capacity degradation. By optimizing the synthetic strategy to reduce the surface exposed sulfur in composite and eliminating the dissolved O_2_ in electrolyte are beneficial to improve the cycling performance of S@CNTs‐50. Furthermore, besides for Zn and S, intense I signal can be also observed at the discharge and charge state (Figure 4h). At the discharged 0.05 V, the peaks at 617 and 628 eV can be assigned to I^−^, while those at 620 and 632 eV can be assigned to the ligand of I_3_^−^. At the charged 1.6 V, peaks at 618 and 630 eV correspond to I_2_. The binding energy change of I at different states also demonstrates the redox reaction of I_2_ additive, during which I_2_ can be functioned as Zn^2+^ ion carrier and reduce the overpotential. During the discharge process, I_2_ is initially reduced to I^−^, which further coordinates with I_2_ to form I_3_^−^. In the charge process, the oxidation reactions of ZnS and I_3_^−^ can take place simultaneously. The good reversibility of I_2_↔I_3_^−^ redox couple suggests that the additive of I_2_ will not be lost and can be stably served as Zn^2+^ ion carrier, as supported by the obvious signal of I_2_ observed in S@CNTs‐50 after 20 cycles (Figure S18, Supporting Information). It is worth noting that water can be also associated with the reactions because the strong O and SO_4_^2−^ signals are also observed in the O1s and S2p XPS spectra. Thus, without additive, the cathode reaction mechanisms can be summarized by Equations (1)–(3)

(1) $$\begin{array}{c} \mathrm{Discharge}:\;S+Zn^{2+}+2e^{-}\to \mathrm{ZnS} \end{array}$$

(2) $$\begin{array}{c} \mathrm{Charge}:\;\mathrm{ZnS}-2e^{-}\to S+Zn^{2+} \end{array}$$

(3) $$\begin{array}{c} 2\mathrm{ZnS}+4H_{2}O-10e^{-}\to 2Zn^{2+}+{\mathrm{SO}}_{4}^{2-}+S+8H^{+} \end{array}$$

With additive, the possible cathode reactions can be expressed by Equations (4)–(7)

(4) $$\begin{array}{c} \mathrm{Discharge}:\;S+Zn^{2+}+I_{2}+4e^{-}\to \mathrm{ZnS}+2I^{-} \end{array}$$

(5) $$\begin{array}{c} I^{-}+I_{2}\to {I_{3}}^{-} \end{array}$$

(6) $$\begin{array}{c} \mathrm{Charge}:\;\mathrm{ZnS}+I_{3}^{-}-3e^{-}\to Zn^{2+}+3/2I_{2}+S \end{array}$$

(7) $$\begin{array}{ccc} & & 2\mathrm{ZnS}+4H_{2}O+I_{3}^{-}-11e^{-}\to 2Zn^{2+}+3/2I_{2}\\ & & \;+\;{\mathrm{SO}}_{4}^{2-}+S+8H^{+} \end{array}$$

In conclusion, we have reported a high energy density and low cost aqueous Zn–S battery with S@CNTs‐50 cathode and Zn foil anode. The influence of pH values, additive of electrolytes and sulfur content on the electrochemical behavior are clarified. In the optimized electrolyte of 1 m Zn(CH_3_COO)_2_ (pH = 6.5) with 0.05 wt% I_2_ as additives and a sulfur content of 50 wt% in S@CNTs composite, S@CNTs‐50 exhibits a very high capacity of 1105 mAh g^−1^ and a maximum energy density of 502 Wh kg^−1^(on the basis of sulfur) at 100 mA g^−1^. Moreover, owing to the I_2_ additive, the kinetics and overpotential of S@CNTs‐50 and Zn cycling can be improved. More importantly, the chemical cost of this aqueous Zn–S battery decreases to $45 kWh^−1^ because of the cheap raw materials. Besides, the combination of ex situ XRD, Raman, XPS, and TEM measurements demonstrate the conversion reaction between S and ZnS. The high energy density and extremely low cost of S@CNTs‐50 demonstrates that it is an attractive cathode for aqueous Zn‐metal based batteries.

## Experimental Section

##### The Synthesis of Carbon Nanotubes Supported S (S@CNTs)

A series of carbon and sulfur composites were prepared by adjusting the mass ratio between CNTs and sulfur. In a typical procedure, the carbon nanotubes and sulfur powers were mixed in a mass ratio of 7:3, 6:4, 5:5, and 4:6, respectively, sealed into four quart tubes in vacuum, and then heated to 160 °C for 16 h. After cooled to room temperature, the quart tubes were cut and the products of S@CNTs were obtained. The samples were named as S@CNTs‐30, S@CNTs‐40, S@CNTs‐50, and S@CNTs‐60, respectively.

##### Materials Characterizations

XRD patterns were collected through an XRD‐7000S diffractometer equipped with a Cu K_*α*1_ Radiation (*λ* = 1.5406 Å). Scanning electron microscopy observations were performed on a JEM7600F microscope at 15 kV. TEM observations were carried out on a JEOL2100 microscope at 200 kV. Raman spectra were recorded on a Renishaw Invia spectrometer by using Ar+ laser of 514.5 nm. The nitrogen adsorption and desorption isotherms were recorded at 77 K by using a Micromeritics ASAP 2020 analyzer and the surface area was calculated using the BET method. XPS spectra were recorded on an Axis Ultra DLD system with a monochromatic Al K_*α*_ X‐ray source.

##### Electrochemical Measurements

CR2016 coin cells were assembled to test the electrochemical properties. The working electrode was prepared by mixing 80 wt% S@CNTs (S@CNTs‐30, S@CNTs‐40, S@CNTs‐50, and S@CNTs‐60) with 10 wt% polytetrafluoroethylene (60 wt% in water) and 10 wt% acetylene black. The obtained mixture was extruded into a thin film by using a rolling machine, punched into 8 mm plates and pressed onto Ti mesh. The mass loading of active material was ≈3–5 mg cm^−2^. A Zn foil was applied as both reference and counter electrode, fiber glass as separator and 1 m Zn(CH_3_COO)_2_ in deionized water with 0.05 wt% I_2_ additive as electrolyte. The dosage of electrolyte in each coin cell was controlled as 60 µL. The battery galvanostatic charge/discharge tests were recorded using a LANHE Battery Tester (China) at room temperature and 55 °C in the potential range of 0.05–1.6 V (vs Zn^2+^/Zn). The capacities were calculated based on the mass of sulfur unless otherwise statement.

## Conflict of Interest

The authors declare no conflict of interest.

## Supporting information

Supporting Information
