# Supplementary material for: A Low Cost Aqueous Zn–S Battery Realizing Ultrahigh Energy Density
Source: Adv Sci (Weinh). 2020 Oct 20;7(23):2000761. doi: 10.1002/advs.202000761 (PMC7709974; doi:10.1002/advs.202000761)
Supplement: Supplementary file 1 — Supporting Information [file ADVS-7-2000761-s001.pdf]

WILEY-VCH

© 2020 Wiley-VCH GmbH

ADVANCED SCIENCE
Open Access

Supporting Information

for *Adv. Sci.*, DOI: 10.1002/advs.202000761

A Low Cost Aqueous Zn-S Battery Realizing Ultrahigh Energy Density

Wei Li, Kangli Wang, and Kai Jiang **

© 2020 Wiley-VCH GmbH

Supporting Information

A low cost aqueous Zn-S battery realizing ultrahigh energy density

Wei Li, Kangli Wang, and Kai Jiang **

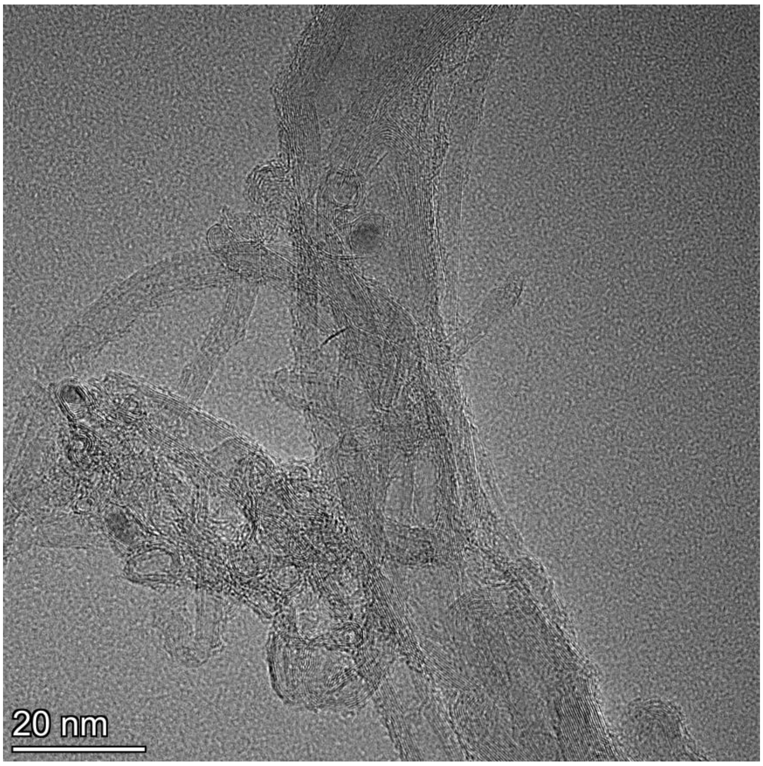

Figure S1. TEM image of S@CNTs-50.

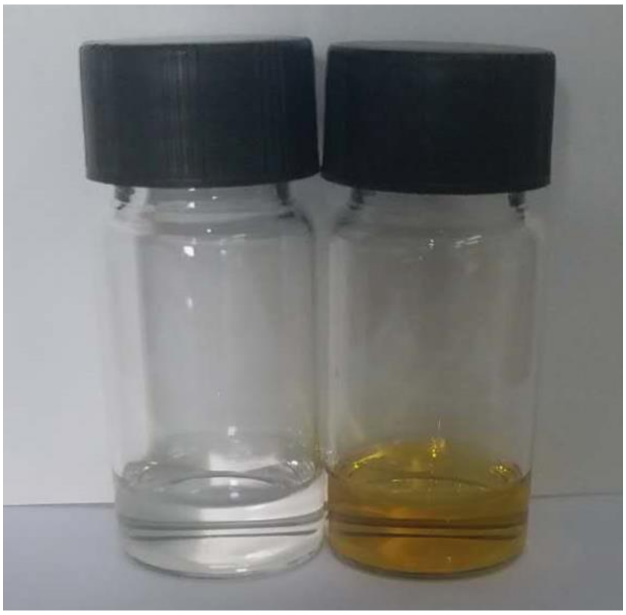

Figure S2. Optical photogrph of 1M $Zn(CH_3COO)_2$ without (left) and with 0.05 wt% I_2(right).

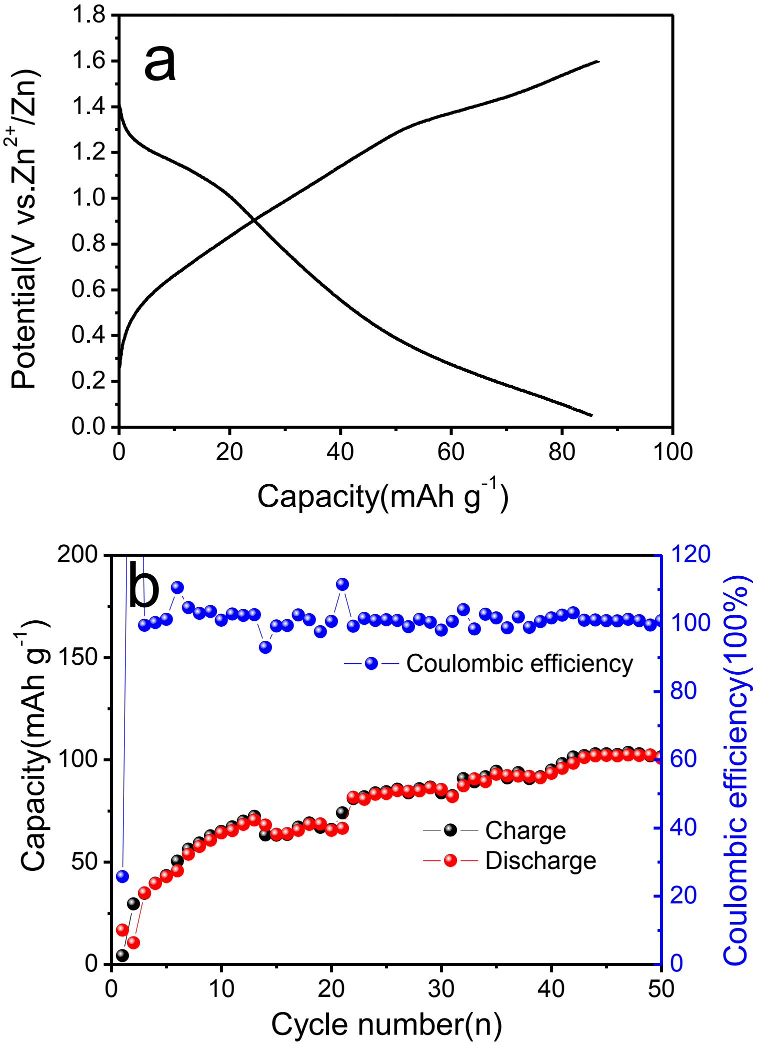

Figure S3. (a) Charge and discharge curves and (b) cycling performance of CNTs electrode in the electrolyte of 1 M $Zn(CH_3COO)_2$+0.05wt% I_2 at a current density of 100 mA g^{-1}.

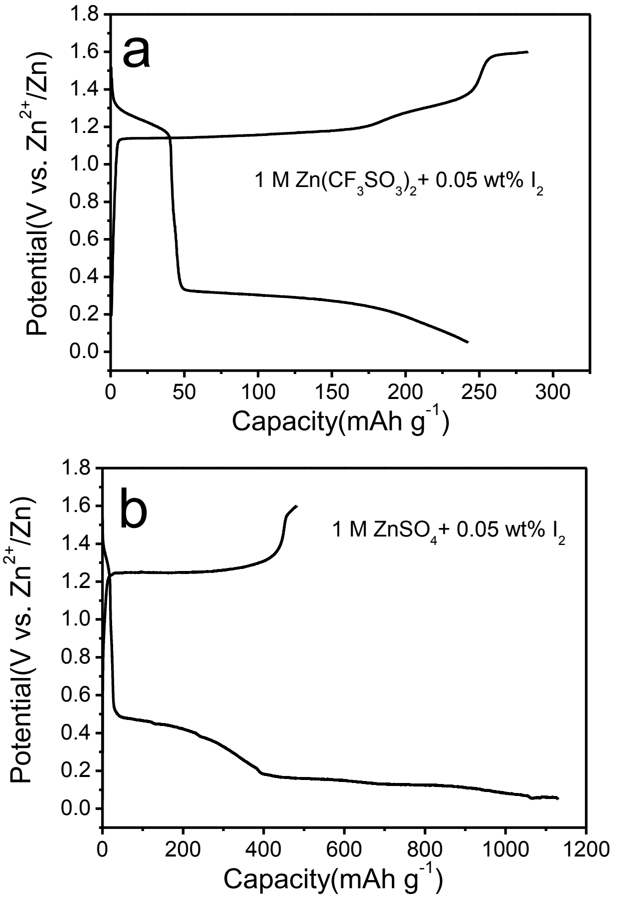

Figure S4. Charge and discharge curves of (a) 1 M $Zn(CF_3SO_3)_2$ and (b) 1M $ZnSO_4$ with I_2 additive.

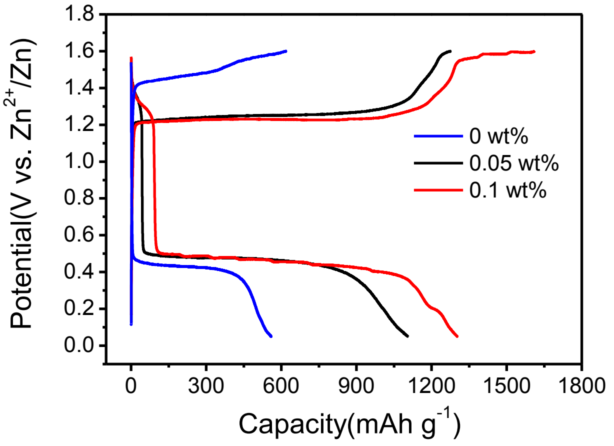

Figure S5. Charge and discharge curves of S@CNTs with different contents additive.

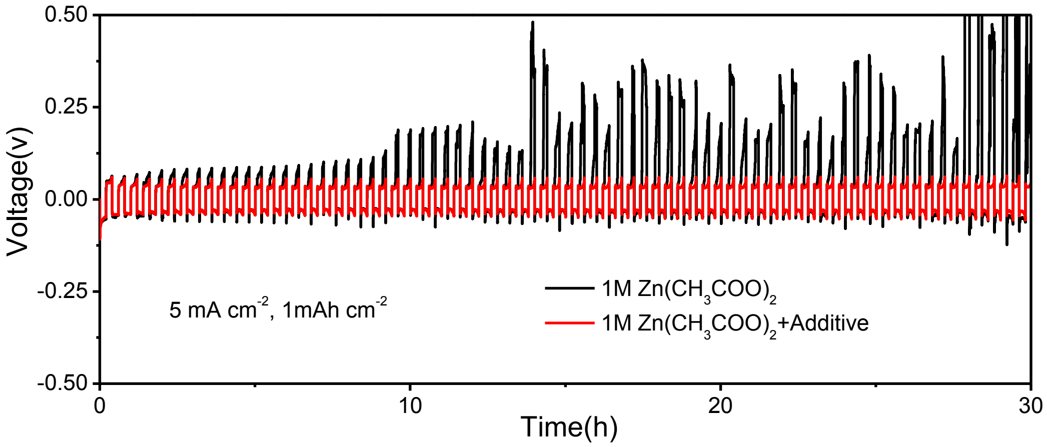

Figure S6. Cycling performance of Zn symmetrical cells in the electrolyte of 1 M $Zn(CH_3COO)_2$ with or without I_2 additive.

Table S1. Cost of electrode materials

Compart	Price (US$/kg)	Ref.
Zn	2	S1
S	0.25	S2
CNTs	20	S3

To simplified the calculation, the cost of 1 M $Zn(CH_3COO)_2$ electrolyte and additive are ignored because of the very lower cost than other counterpart. Since the energy density of S@CNTs is 502 Wh $kg^{-1}$and the total cost of Zn, S and CNTs are 22.25 $/kg, the cost of can be calculated to be 1000×(2+0.25+20)/502= USD $ 45/kWh.

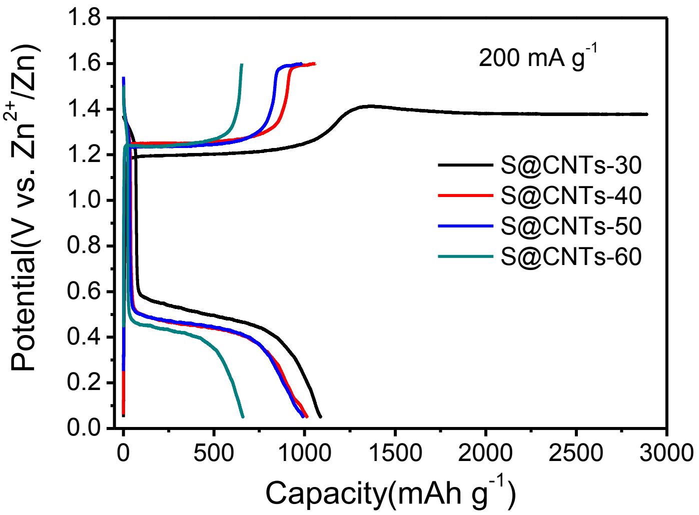

Figure S7. Charge and discharge curves of four electrodes of S@CNTs with different sulfur contents at 200 mA g^{-1} in the electrolyte of 1 M $Zn(CH_3COO)_2$ with I_2 additive.

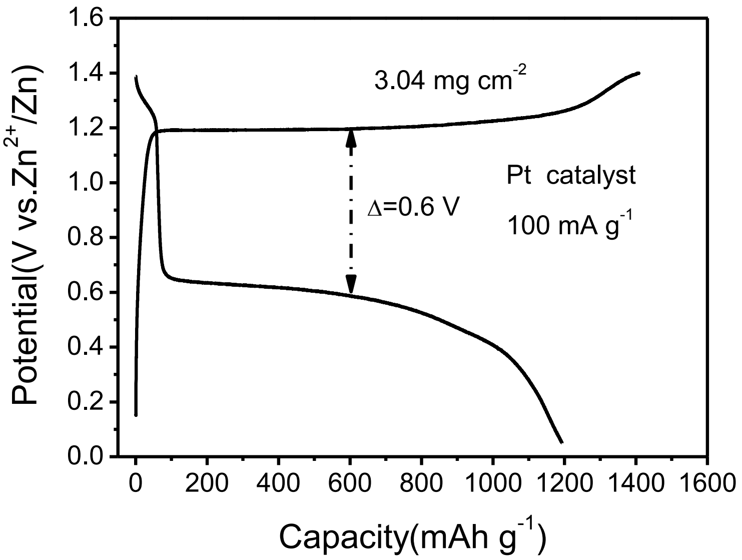

Figure S8. Charge and discharge curve of S@CNTs with 5wt% Pt at 100 mA g^{-1} with a sulfur loading of 3.04 mg cm^{-2}.

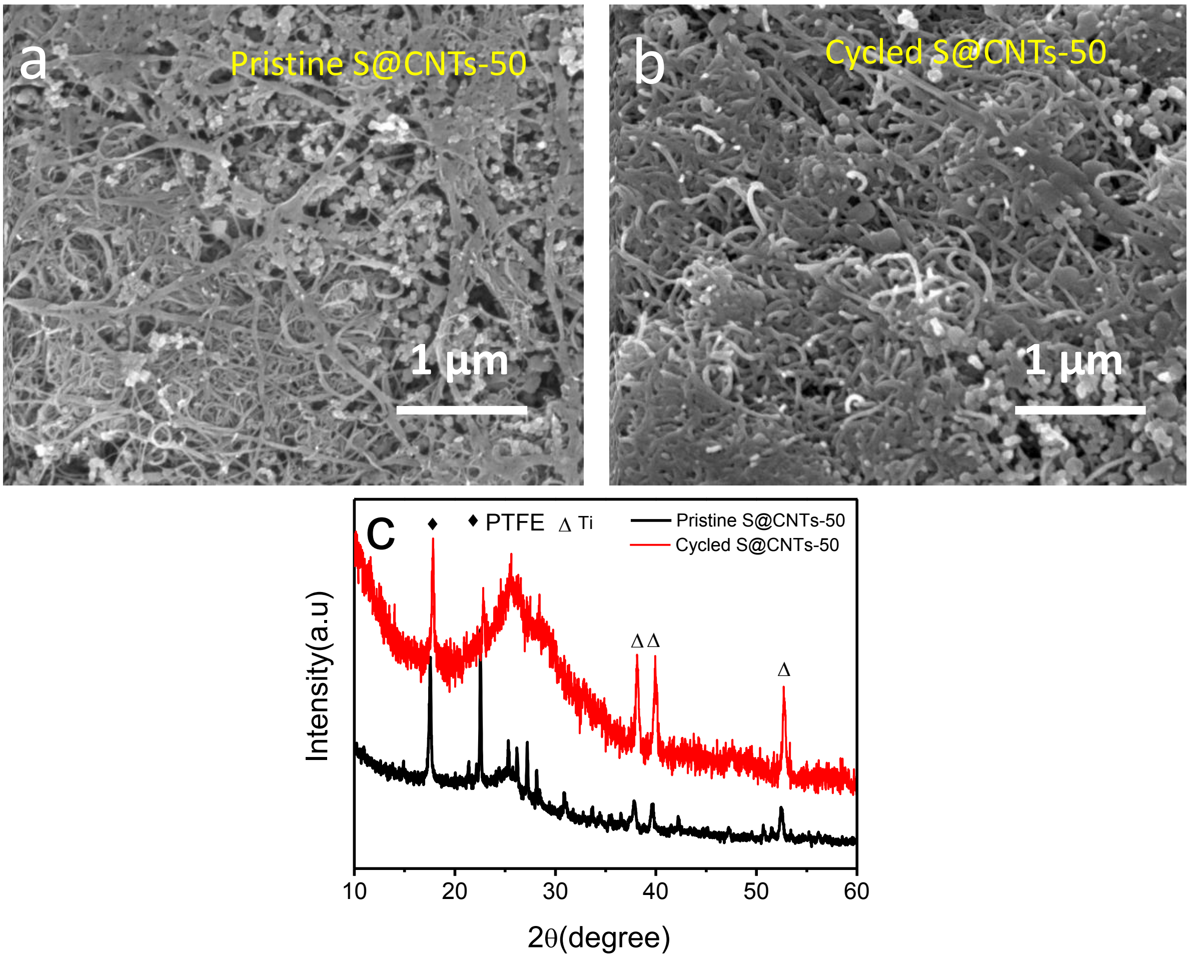

Figure S9. SEM images of S@CNTs-50 (a) before and (b) after 160 cycles, (c) XRD patterns of pristine and cycled S@CNTs-50 electrodes.

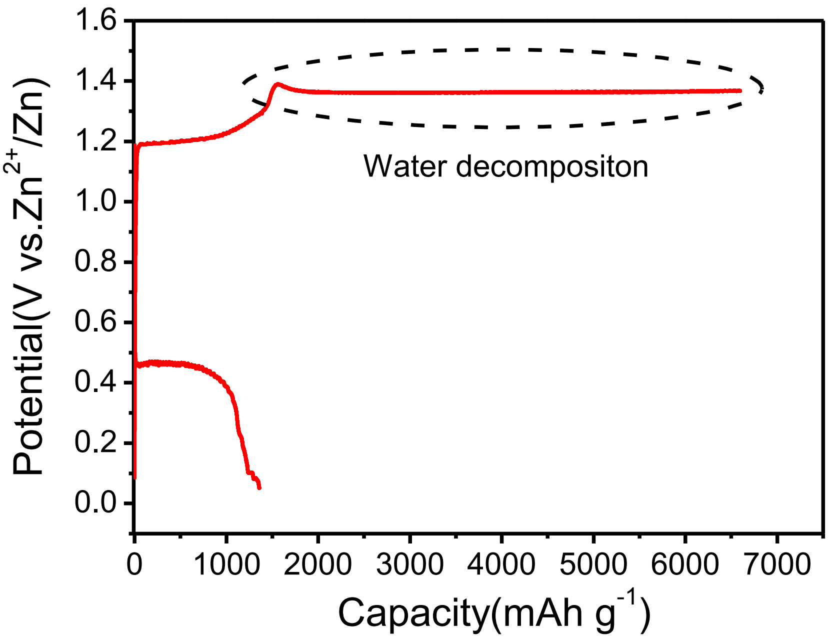

Figure S10. Charge and discharge curves of S@CNTs-50 at a current density of 20 mA g^{-1}.

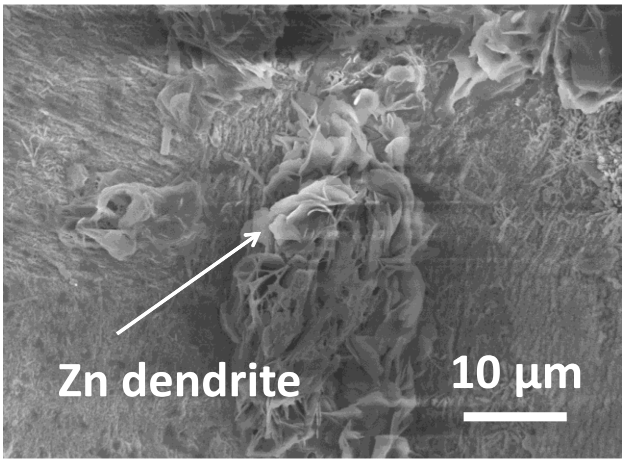

Figure S11. SEM image of cycled Zn.

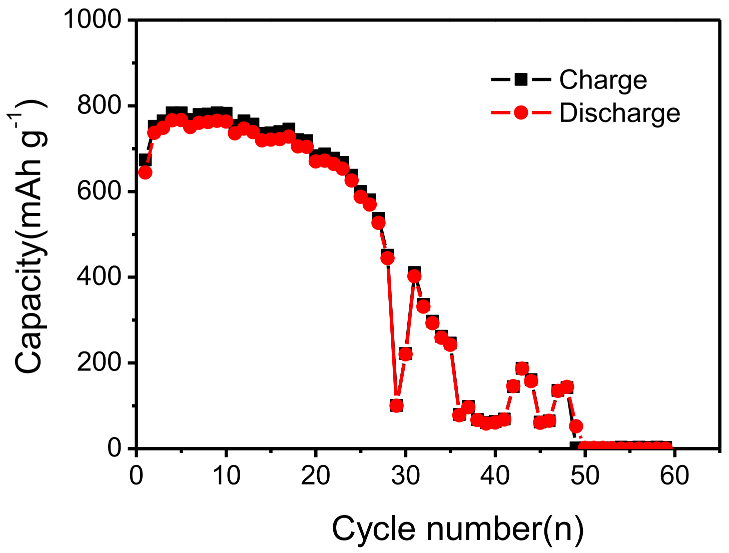

Figure S12. Cycling of S@CNTs-50 at a current density of 1000 mA g^{-1}.

Table S2. Comparison of S@CNTs-50 with other reported cathode for aqueous Zn-based batteries.

Materials	Voltage (V)	Capacity (mAh g^{-1})	Energy density (Wh kg^{-1})	Current density (A g^{-1})	Cycling	Capacity retention	Ref.
$Zn_{0.25}V_2O_5{\cdot}nH_2O$	0.7	282	250	2.4	1000	82	*Nat. Energy* 2016, 1, 16119
$Na_3V_2(PO_4)_2F_3$	1.62	65	100	1	4000	95%	*Energy Storage Mater*. 2018, 15,14
$ZnMn_2O_4$	1.4	150	202	0.5	500	94%	*J. Am. Chem. Soc*. 2016, 138, 12894
ZnHCF	1.7	65	100	0.06	100	76	*Adv. Energy Mater*. 2015, *5*, 1400930
$Na_3V_2(PO_4)_3$	1.1	97	100	0.05	100	74%	*Nano Energy* 2016, 25, 211
VS_2	0.6	190	123	0.5	200	82%	*Adv. Energy Mater*. 2017, *7*, 1601920
$Zn_3V_2O_7(OH)_2{\cdot}2H_2O$	0.7	200	150	0.2	300	68%	*Adv. Mater*. 2017, 1705580
$K_2V_6O_{16}{\cdot}2.7H_2O$	0.8	230	172	6	500	82	*J. Mater. Chem. A*, 2018, 6, 15530
$Na_2V_6O_{16}{\cdot}3H_2O$	0.8	361	287	14.4	1000	80	*Nano Lett*. 2018, 18, 2402
$Zn_2(OH)VO_4$	0.8	140	200	4	2000	89%	*Adv. Mater*. 2018, 1803181
$V_2O_5{\cdot}nH_2O$	0.7	372	290	6	900	71%	*Adv. Mater*. 2017, 1703725
$Zn_2V_2O_7$	0.8	200	166	4	1000	85%	*J. Mater. Chem. A* 2018, 6, 3850
PTO	0.8	336	186.7	3	1000	70	*Angew. Chem. Int. Ed*. 2018, 57, 11737
VO_2	0.6	280	160	4	1000	99%	*Energy Storage Mater*. 2019, 17, 143
$Na_3V_2(PO_4)_3$	1.23	114	140	0.5	200	75	*Nano Energy* 2019, 58, 492
I_2@C-50	1.2	210	237	0.1	50	87%	*J. Mater. Chem. A* 2020, 8, 3785
S@CNTs-50	0.5	1105	502	2	300	76%	This work

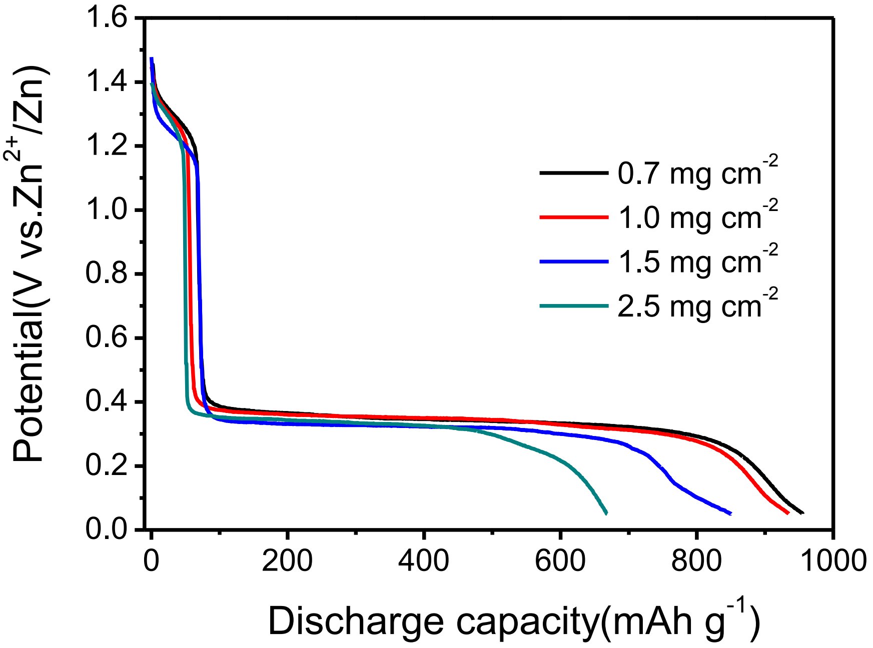

Figure S13. Discharge curves of S@CNTs-50 with different sulfur loadings at a current density of 1000 mA g^{-1}.

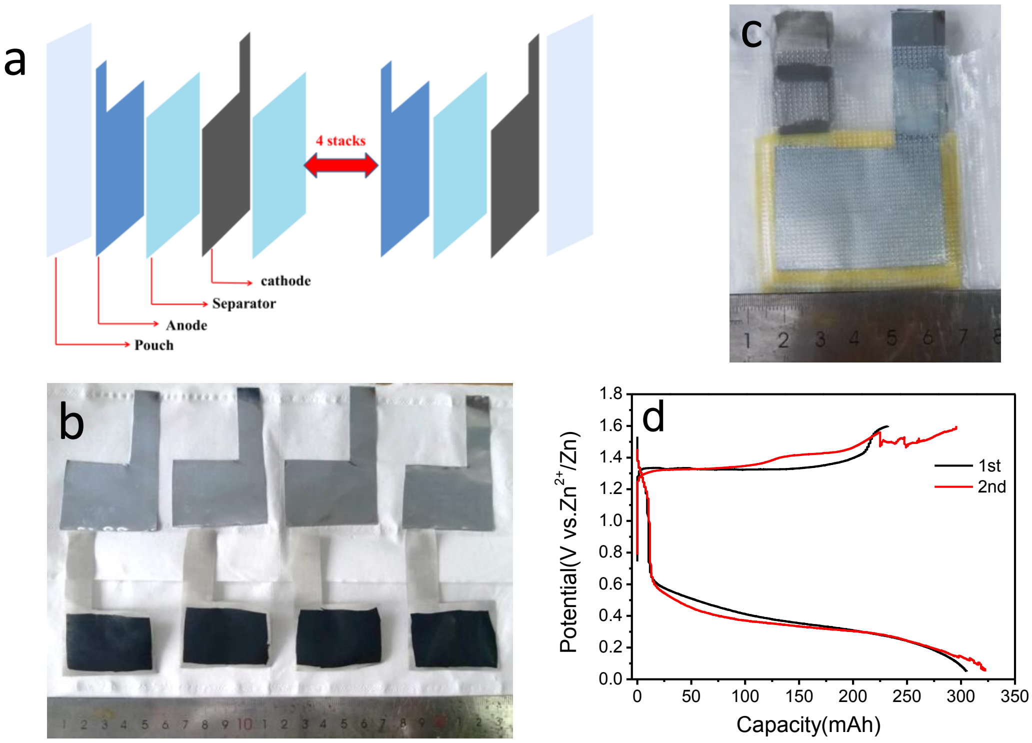

Figure S14. (a) Structural illustration, (b) optical photograph of anodes and cathodes, (c) packing battery with four anode-separator-cathode stacks with a designed capacity of 0.35 Ah, (d) charge and discharge curves of packing battery at a current density of 100 mA g^{-1}.

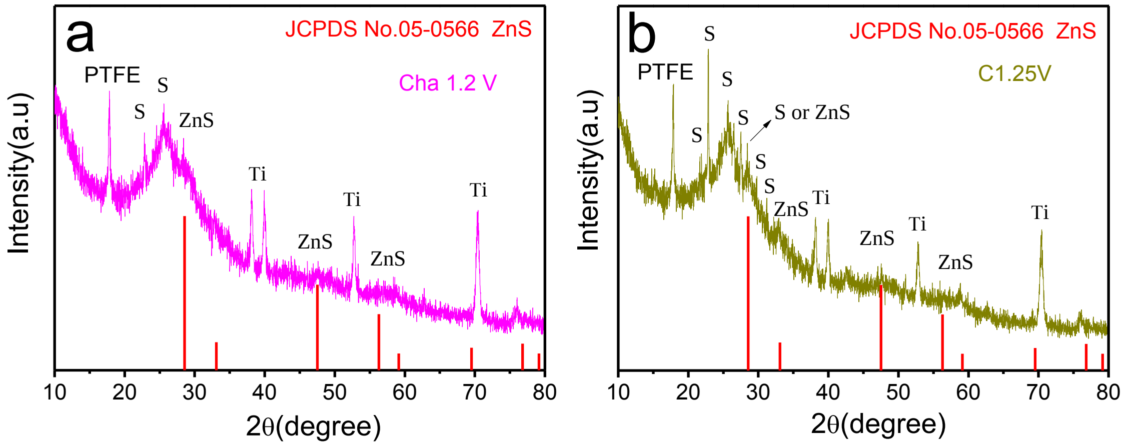

Figure S15. XRD patterns of S@CNTs-50 at charged (a)1.2 V and (b) 1.25 V.

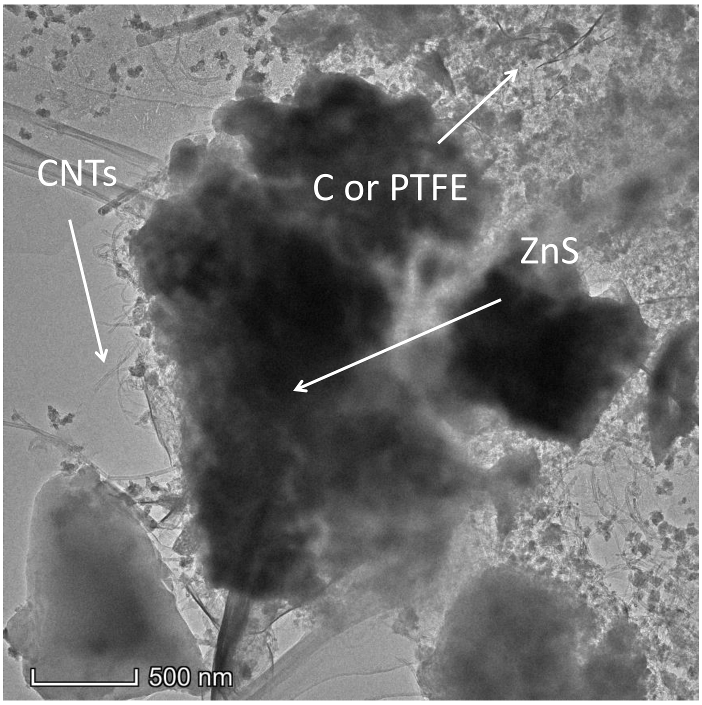

Figure S16. TEM image of S@CNTs-50 at fully dischared state.

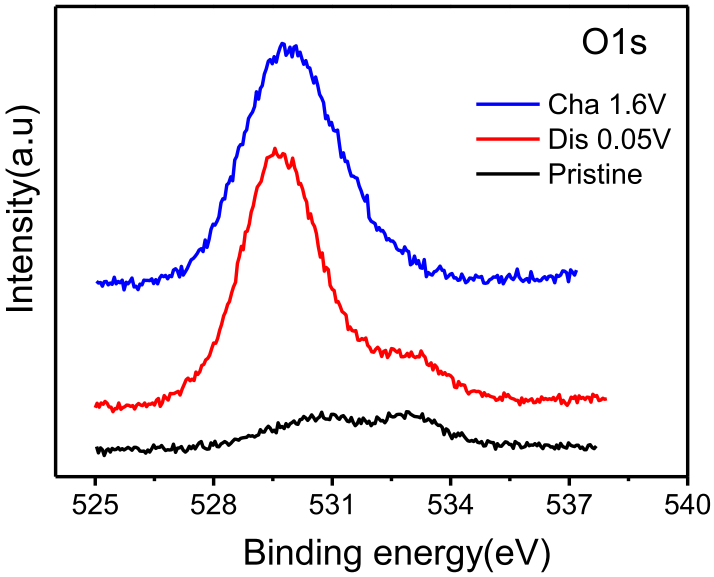

Figure S17. XPS of O1s at the pristine, fully discharged state of 0.05V and fully charged state of 1.6 V sate.

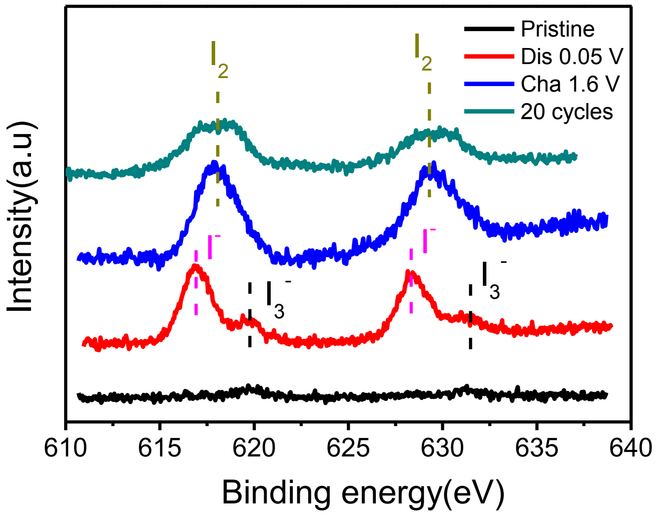

Figure S18. XPS of I3d of S@CNTs-50 at the pristine, fully discharged state of 0.05V, fully charged state of 1.6 V sate, and after 20 cycles。
